# Examining the effect of safety climate on accident risk through job stress: a path analysis

**DOI:** 10.1186/s40359-023-01133-2

**Published:** 2023-03-30

**Authors:** Amir Hossein Khoshakhlagh, Saleh Al Sulaie, Saeid Yazdanirad, JeeWoong Park

**Affiliations:** 1 Department of Occupational Health Engineering, Faculty of Health, Kashan University of Medical Sciences, Kashan, Iran; 2 Students’ Scientific Research Center, Tehran University of Medical Sciences, Tehran, Iran; 3 Department of Industrial Engineering, College of Engineering in Al-Qunfudah, Umm Al-Qura University, Makkah, 21955 Saudi Arabia; 4 Social Determinants of Health Research Center, Shahrekord University of Medical Sciences, Shahrekord, Iran; 5 School of Health, Shahrekord University of Medical Sciences, Shahrekord, Iran; 6 Department of Civil and Environmental Engineering and Construction, University of Nevada, Las Vegas, USA

**Keywords:** Job stress, Workplace, Safety climate, Accident risk, Structural equation modeling

## Abstract

**Background:**

Job stress is a probable mediator of the relationship between safety climate and accident occurrence. To demonstrate this, this study investigates the relationship between safety climate, job stress, and accident risk using a large number of surveys. The study will use structural equation modeling (SEM) to analyze the data collected from the surveys to examine the effect of safety climate on accident risk through job stress.

**Methods:**

The study is a cross-sectional study that was conducted on 1,530 male workers of a petrochemical company. The subjects were asked to complete several questionnaires during rest periods, which included demographic information, the Nordic safety climate questionnaire (NOSACQ-50), and the generic job stress questionnaire (GJSQ). Additionally, data on the frequency and intensity of accidents among participants were gathered from the health unit of the company. Path analysis was conducted by structural equation modeling (SEM) in Analysis of Moment Structures (AMOS) software.

**Results:**

The results revealed that the latent variable of safety climate with an effect coefficient of – 0.112 did not have a direct effect on accident risk (P = 0.343). However, safety climate with an effect coefficient of − 0.633 had an indirect effect on accident risk through job stress (P < 0.001). The total score of job stress had a significant direct effect (0.649) on accident risk (P < 0.001). Among the dimensions of safety climate, the variables of management’s safety priority, commitment, and competence (− 0.108) and workers’ safety commitment (− 0.107) had the highest indirect effect coefficients on accident risk. Among the dimensions of job stress, the highest indirect effects belonged to the variables of conflict at work (0.636), physical environment (0.631), and workload and responsibility (0.631), respectively.

**Conclusion:**

The results of the study revealed that job stress mediates the relationship between safety climate and accident risk. This finding suggests that organizations can potentially decrease accidents in industries by addressing and managing job stress in the workplace.

## Background

Occupational accidents pose a significant threat to human health, the economy, society, and the environment [1]. These accidents are often quantified using two metrics: frequency and intensity. Frequency refers to the number of accidents that occur within a specified period, while intensity is the level of consequences resulting from an accident [2]. According to the international labor organization (ILO), there are approximately 2.78 million annual cases of work-related accidents or diseases, which carry a considerable financial burden of over 1.25 trillion dollars [3]. Furthermore, occupational accidents are among the leading causes of mortality in many countries. For example, based on data ranging from 2007 to 2016, occupational accidents and injuries were ranked as the third cause of mortality in the world and the second in Iran [3].

Various factors can increase the risk of accidents and affect their occurrence. Two of the most important factors are workplace physical conditions and human behavior [4]. Research shows that human behavior plays a greater role than workplace physical conditions, 60 to 90% of accidents are directly caused by human behavior [5].

Safety climate is a crucial factor that can impact worker behavior in the workplace. Safety climate is defined as the shared perceptions among employees about the importance of safety in their work environment [6]. It is a multi-dimensional factor, including aspects such as commitment to safety at individual and management levels, safety communication, and safety system in place during operations [7]. The results of a study performed by Ajslev et al. show that there is a clear relationship between safety climate and accident occurrence [8]. Other studies have shown that a positive safety climate is associated with safer behaviors and reduced accidents [9, 10]. It also has a positive effect on safety outcomes such as compliance, participation behaviors, accidents, and injuries [11, 12]. Furthermore, a good safety climate can mediate the relationship between organizational climate and the safety function of workers, with several studies exploring the relationship between safety climate and accident occurrence [8–12].

Job stress is also associated with the behavior and performance of employees. This type of stress is typically caused by workplace conditions that negatively affect the well-being of workers [13]. A study by Leung et al. [14] showed that job stress is associated with unsafe behaviors and thus affects the occurrence of accidents. It can also lead to decreased concentration, distraction, memory impairment, work hesitation, and decision-making power in the workers [15]. Therefore, job stress is a major contributor to occupational accidents and injuries, accounting for 37% of such accidents [16]. Additionally, Kim et al. [17] showed a direct relationship between job stress and injury occurrences among firefighters.

Literature suggests that job stress is a probable mediator between safety climate and accident occurrence. Safety climate regulates/controls work demand based on resource interaction and expected job stress [18]. An unbalanced workload per personal capability and work productivity increase job stress [19]. We can also view job stress as being associated with other aspects, such as job control, conflict at work, job satisfaction, mental demand, physical environment, social support, workload, and responsibility [20] in an indirect relationship. When there is a great imbalance between personal capabilities and job demands, work-related stress is created in humans [19]. Job stress is also associated with other agents, such as job control, conflict at work, job satisfaction, mental demand, physical environment, social support, workload, and responsibility [20], which may be indirectly related to safety climate, injuries, and accidents.

Despite the association among many factors, there still is lacking understanding of the effect of safety climate on the risk of accidents. In this regard, the present study aims to investigate the effect of safety climate on accident risk through job stress. The study plans to use path analysis to further investigate this relationship based on all factors discussed in this literature.

## Methods

### Participants

This cross-sectional study, conducted in 2019, recruited participants from a large-scale petrochemical company in Iran. A total of 4,621 workers from various departments including technical, electrical, machinery, maintenance, mechanical, welding, turning, and supervision were considered for the study. Then, a random sample of 2,100 individuals was selected, with the number of individuals chosen from each department being proportional to the number of workers in that department. Following this, the medical records of these individuals were reviewed, and those who met the inclusion criteria of having work experience higher than one year and having literacy were entered into the study. The included individuals were 1,912. Exclusion criteria included the lack of cooperation and the inability to complete the questionnaires. Out of the 1,912 workers who met the inclusion criteria, 1,742 accepted to participate in the study and completed the questionnaires, resulting in a sample of 1,530 subjects.

### Data collection

The study protocol was reviewed and approved by the medical ethics committee of Tehran University of Medical Sciences, and all study procedures were conducted in accordance with the ethical code IR.TUMS.VCR.REC.1398.558. Data collection took place at the participants’ workplaces, where researchers provided a general overview of the study and trained them on how to complete the questionnaires. All participants voluntarily gave informed consent and were assured anonymity during the survey. All participants were asked to complete paper questionnaires in the presence of the researchers during their free time. The questionnaires included demographical information, the Nordic safety climate questionnaire (NOSACQ-50), the NIOSH generic job stress questionnaire (GJSQ), and an accident history form.

### Tools

#### Demographical information questionnaire

The demographic information questionnaire included several questions about age, education, marital status, job type, work department, work experience, and habits of the workers.

#### Nordic safety climate questionnaire (NOSACQ-50)

The Nordic safety climate questionnaire is a valid instrument that evaluates individuals’ perceptions of safety climate. The questionnaire was developed by a team of specialists from various Nordic countries, such as Denmark, Norway, Iceland, Finland, and Sweden, in 2011 [21]. This instrument has been used in several studies. For instance, Fargnoli and Lombardi [21, 22] studied safety climate in agricultural activities using the NOSACQ-50, and Marin et al. [23] used this questionnaire to evaluate the perception of safety climate among construction personnel.

The NOSACQ-50 consists of 50 questions that assess seven dimensions of safety climate, including management’s safety priority, commitment, and competence (9 items), management’s safety empowerment (7 items), management’s safety justice (6 items), workers’ safety commitment (6 items), workers’ safety priority and risk non-acceptance (7 items), safety communication, learning, and trust in co-workers’ safety competence (8 items), and workers’ trust in the efficacy of safety systems (7 items) [21]. The participants answered the questions on a one-to-four Likert scale, including ‘strongly disagree,’ ‘disagree,’ ‘agree,’ and ‘strongly agree.’ The study then further evaluated the data in relation to the employees of the participating company. The total safety climate score was computed as the mean value of the scores for the seven dimensions.

A Persian version of the questionnaire was developed by Yousefi et al. [24]. This has been verified for its validity, completeness, and reliability for Cronbach’s alpha coefficient of this version was reported by 0.94 [24].

#### NIOSH generic job stress questionnaire (GJSQ)

The NIOSH GJSQ, developed by the US National Institute for Occupational Safety and Health (NIOSH), has been adopted/used by several studies. Researchers have used it to study the occupational stress of nurses [25] and the job stress dimensions of Japanese employees working in hospitals, transportation, manufacturing, information technology, pharmaceutical, and service industries [26].

Our study applied the NIOSH GJSQ to assess various job stress dimensions, including background information (7 items), conflict at work (16 items), job control (16 items), employment opportunities (4 items), somatic complaints (17 items), general job information (12 items), health condition (24 items), self-esteem (10 items), job requirements (10 items), job satisfaction (4 items), mental demands (5 items), non-work activities (7 items), depression (20 items), physical environment (10 items), problems at work (6 items), social support (12 items), work hazards (5 items), work limitations (5 items), workload and responsibility (11 items), role conflict and ambiguity (14 items), and job future ambiguity (5 items).

The responses are in the form of ‘yes or no,‘ ‘false or true,‘ or a one-to-five Likert scale [27]. The dimensions with qualitative and non-Likert answers including background information, general job information, health conditions, non-work activities, and work limitations were excluded from further consideration. The total score of each dimension was also computed as the mean value of the scores of its questions.

The study used a Persian-translated version of the questionnaire, validated by Kazronian et al. (2013), and found high reliability with a Cronbach’s alpha coefficient and intra-cluster correlation of greater than 0.70 [20].

#### Accident history form

Data about the intensity and frequency of accidents collected from the participants were processed through the health unit of the petrochemical company. The score of accident risk was computed as a multiplication of the severity and frequency of the accident.

### Data analysis

Data were analyzed using the statistical package for the social sciences (SPSS) software version 26. The normality of variables was confirmed through the examination of skewness and kurtosis curves. Given that the statistical distribution of all parameters was normal, correlation coefficients were calculated by the Pearson test. A theoretical model was then described in AMOS software for path analysis. Path Analysis is a causal modeling approach to exploring the correlations within a defined network. This study further used Structural Equation Modeling (SEM), which is a strong multivariate analysis technique, widely used in various sciences [28]. It fulfills a flexible framework to develop and analyze the complex relationships among multiple variables [29]. Absolute, comparative, and normed fit indices were used to evaluate the fitness of this model, and the direct and indirect effect coefficients of the safety climate and job stress dimensions were computed. The significance level used was 0.05, meaning the test result was acceptable.

## Results

The average age of the participants was 36.77 years with a standard deviation of 7.53, and their average work experience was 10.15 years with a standard deviation of 5.01. Tables 1 and 2 provide more information on demographic variables and study variables, which are discussed in detail in the discussion section. Additionally, the accident score ranged from 1 to 30.

**Table 1:** Descriptive statistics related to demographic variables

Demographic variables		Frequency	Valid percent (%)
Age (years)	Lower than 30	226	14.8
	30–40	688	45.0
	41–50	537	35.1
	Higher than 50	79	5.20
Education degree	Under diploma	247	16.1
	Diploma	687	44.9
	Associate degree	460	30.1
	Bachelor degree	127	8.3
	Master degree and higher	9	0.6
Career length (years)	1–5	240	15.7
	6–10	608	39.7
	11–15	470	30.7
	Higher than 15	212	13.9
Marital status	Single	238	15.6
	Married	1,292	84.4
Accident history	Yes	1,139	74.4
	No	391	25.6

* Sample size was 1,530 participants

**Table 2:** The descriptive information of the studied variables/dimensions

Dimension		Range (Minimum - maximum)	Mean	Std. Deviation
Safety climate	Management’s safety priority, commitment, and competence	1.00–4.00	1.94	0.78
	Management’s safety empowerment	1.14–3.86	2.03	0.71
	Management’s safety justice	1.00–4.00	2.09	0.67
	Workers’ safety commitment	1.00–4.00	1.97	0.79
	Workers’ safety priority and risk non-acceptance	1.14–4.00	1.92	0.77
	Safety communication, learning, and trust in co-workers’ safety competence	1.13–3.88	2.07	0.68
	Workers’ trust in the efficacy of safety systems	1.00–3.86	1.97	0.78
	Total score	1.24–3.77	2.00	0.70
Job stress	Job control	1.00–4.93	2.22	1.07
	Conflict at work	1.00–4.75	2.30	1.07
	Employment opportunities	1.00–5.00	3.80	1.10
	Somatic complaints	1.00–5.00	3.50	1.04
	Self-esteem	1.00–4.70	2.23	1.06
	Job requirements	1.00–5.00	3.79	1.12
	Job satisfaction	1.25–3.25	3.34	0.97
	Mental demands	1.00–4.00	3.87	1.12
	Depression	0.00–3.00	2.60	1.00
	Physical environment	1.00–2.00	3.74	1.38
	Problems at work	1.33–4.17	2.68	0.56
	Social support	1.00–5.00	3.73	0.97
	Work hazard	1.00–5.00	3.64	1.12
	Workload and responsibility	1.00–4.86	3.71	1.03
	Role conflict and ambiguity	1.50–6.73	3.28	0.94
	Job future ambiguity	1.20–5.00	2.36	1.06
	Total score	2.27–3.33	2.98	0.27
	Frequency	1.00–5.00	2.27	1.85
Accident	Intensity	1.00–6.00	2.84	1.61
	Risk score	1.00–30.0	12.4	8.43

* Sample size was equal to 1,530 persons

Table 3 presents the correlation between two variables (i.e., job stress and safety climate) and accident risk. The correlations were found to be significant (P < 0.01) across all dimensions of safety climate and job stress with accident risk. The two highest correlations with accident risk in the safety climate dimensions were management’s safety justice (-0.505) and workers’ safety priority and risk non-acceptance (-0.501) Among the job stress dimensions, the most significant correlations were related to the variables of the physical environment (0.541), Conflict at work (0.528), and workload and responsibility (0.528), respectively. The correlation coefficients between the total scores of safety climate and job stress and the score of accident risk also were – 0.518 and 0.523, respectively.

**Table 3:** Correlation coefficients of two dimensions (i.e., job stress and safety climate) with accident risk

Variable	X26	Variable	X26
X1	-0.497^**^	X14	0.526^**^
X2	-0.496^**^	X15	-0.471^**^
X3	-0.505^**^	X16	0.510^**^
X4	-0.495^**^	X17	0.476^**^
X5	-0.501^**^	X18	0.541^**^
X6	-0.487^**^	X19	0.377^**^
X7	-0.482^**^	X20	-0.500^**^
X8	-0.518^**^	X21	0.517^**^
X9	-0.520^**^	X22	0.528^**^
X10	0.528^**^	X23	0.520^**^
X11	-0.492^**^	X24	0.515^**^
X12	0.507^**^	X25	0.523^**^
X13	-0.507^**^		

X1 = Management’s safety priority, commitment, and competence; X2 = Management’s safety empowerment; X3 = Management’s safety justice; X4 = Workers’ safety commitment; X5 = Workers’ safety priority and risk non-acceptance; X6 = Safety communication, learning, and trust in co-workers’ safety competence; X7 = Workers’ trust in the efficacy of safety systems; X8 = Total safety climate; X9 = Job control; X10 = Conflict at work; X11 = Employment opportunities; X12 = Somatic complaints; X13 = Self-esteem; X14 = Job requirements; X15 = Job satisfaction; X16 = Mental demands; X17 = Depression; X18 = Physical environment; X19 = Problems at work; X20 = Social support; X21 = Work hazard; X22 = Workload and responsibility; X23 = Role conflict and ambiguity; X24 = Job future ambiguity; X25 = Total job stress; X26 = Accident risk; N = 1530

**Fig. 1:**
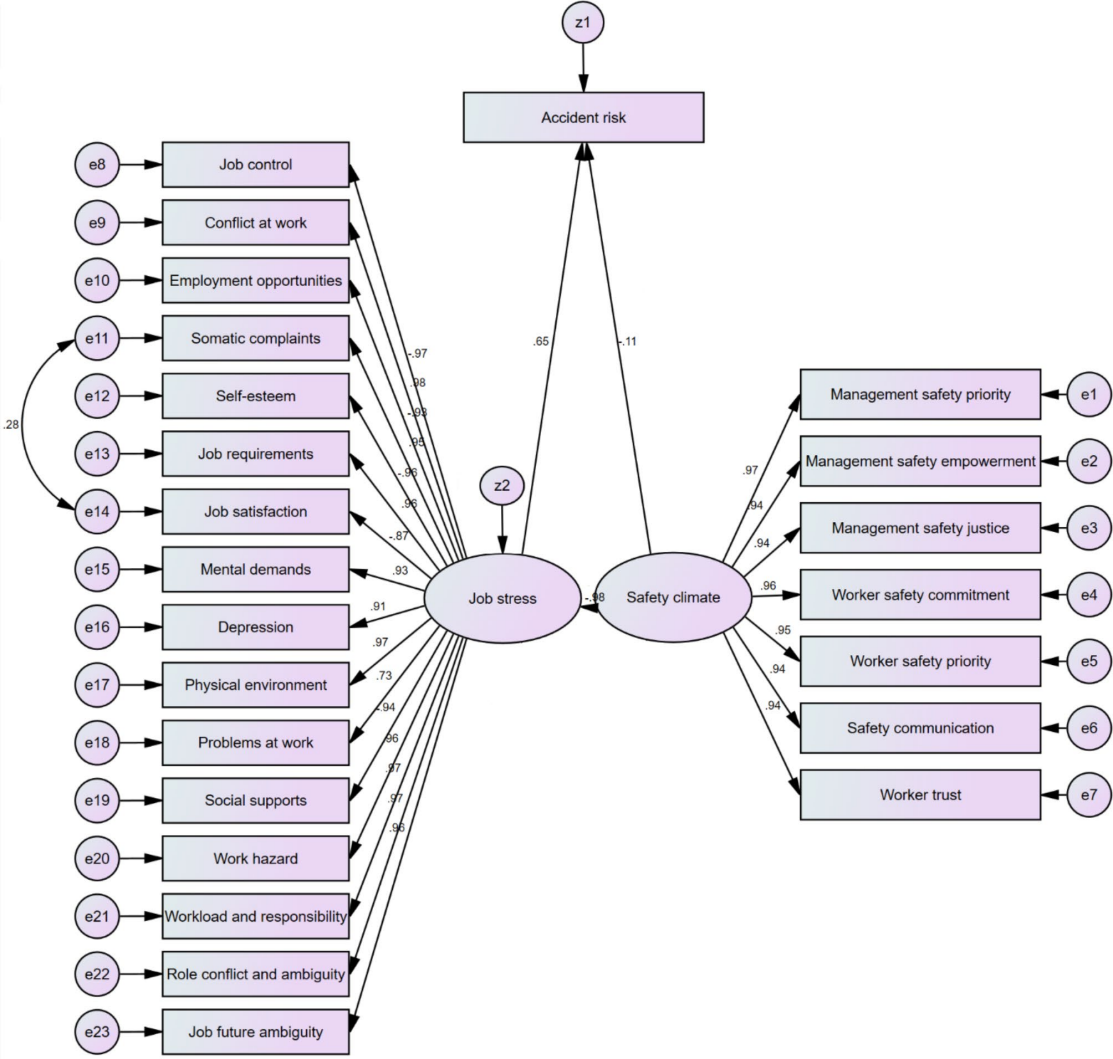
Model designed for examination of the relations between the studied variables

The study examines the relationships among the studied variables using the model presented in Fig. 1. Table 4 represents the goodness-of-fit indices of the model, which demonstrated that the model fits the data well. The arrows in Fig. 1 indicate the direction of the relationship between variables, and the values on the arrows show their direct effect coefficients. The results show that the latent variable of safety climate with the effect coefficients of − 0.112 does not have a direct effect on accident risk (P = 0.343). However, this variable with the effect coefficients of − 0.633 has an indirect effect through job stress (P < 0.001). Additionally, the total score of job stress has a significant direct effect (0.649) on accident risk (P < 0.001).

**Table 4:** The fit indices of the model

Indices	Name	Fitness	Obtained value
Absolute fitness indices	Goodness-of-fit index (GFI)	> 0.9	0.922
	Adjusted goodness-of-fit index (AGFI)	> 0.9	0.912
Comparative fitness indices	Normed fit index (NFI)	> 0.9	0.908
	Comparative fit index (CFI)	> 0.9	0.942
	Incremental fit index (IFI)	0–1	0.947
Normed fit index	Root mean squared error of approximation (RMSEA)	< 0.1	0.065
	Normed Chi-square (X2/df)	1–3	2.070

Table 5 reports the effect coefficients of the job stress and safety climate dimensions on accident risk. Of the safety climate dimensions, the variables of management’s safety priority, commitment, and competence (− 0.108) and workers’ safety commitment (− 0.107) are ranked as the top two indirect effect coefficients on accident risk. Among the job stress dimensions, the highest indirect effects were the variables of conflict at work (0.636), physical environment (0.631), workload and responsibility (0.631), and job control (− 0.630), respectively, while most were high.

**Table 5:** The effect coefficients of the job stress and safety climate dimensions on accident risk

Variable		Direct effect	Indirect effect
Safety climate dimensions	Management’s safety priority, commitment, and competence	0.967	-0.108
	Management’s safety empowerment	0.944	-0.106
	Management’s safety justice	0.942	-0.106
	Workers’ safety commitment	0.958	-0.107
	Workers’ safety priority and risk non-acceptance	0.948	-0.106
	Safety communication, learning, and trust in co-workers’ safety competence	0.940	-0.105
	Workers’ trust in the efficacy of safety systems	0.939	-0.105
	Total score	-0.112	-
Job stress dimensions	Job control	-0.971	-0.630
	Conflict at work	0.980	0.636
	Employment opportunities	-0.932	-0.605
	Somatic complaints	0.948	0.615
	Self-esteem	-0.956	0.620
	Job requirements	0.963	0.625
	Job satisfaction	-0.873	-0.567
	Mental demands	0.932	0.605
	Depression	0.911	0.591
	Physical environment	0.973	0.631
	Problems at work	0.735	0.477
	Social support	-0.937	0.608
	Work hazard	0.956	0.620
	Workload and responsibility	0.972	0.631
	Role conflict and ambiguity	0.966	0.627
	Job future ambiguity	0.958	0.622
	Total score	0.649	-

* Sample size was equal to 1,539 persons

## Discussion

This study examined the relationships between safety climate, job stress, and accident risk. The results found that all dimensions of safety climate and job stress had significant correlations with accident risk, and a path analysis supported these findings. One of the interesting results from the path analysis was that safety climate did not directly affect accident risk but indirectly impacted accident risk through job stress.

Previous studies show the effect of safety climate on accidents. Kalteh et al. performed a systematic review to investigate the relationship between safety culture and safety climate and safety performance. They concluded that improving the safety climate can be effective in reducing accidents and increasing safe performance [30]. The results of a study performed by Probst et al. also showed that a poorer organizational safety climate was associated with the under-reporting of accidents and inappropriate safety enforcement [31]. The results of a study conducted by Neal and Griffin indicated that safety climate can affect safety motivation, safety behavior, and accidents at the work [10]. Nielsen et al. also concluded that changes in the safety climate can lead to changes in the accident rate in work environments [32]. The effect of safety climate on accident rate can be regulated by various factors. However, what is important in the present study compared to previous studies is the effect of safety climate on the rate of accidents through job stress. The results of previous studies show job stress has a significant impact on accidents. Barkhordari et al. concluded that occupational stress can lead to personal failure and carelessness and increase unsafe acts and accidents [33]. The results of a study performed by Mohammadfam et al. showed that there are significant direct relationships between job stress and unsafe acts and accidents [34]. On the other hand, the results of a previous study indicate safety climate can affect workers’ job stress. The study by Akbolat et al. revealed the effect of workplace safety climate on job stress through the moderating effect of psychological well-being [35]. Idris et al. also concluded that safety climate had a significant effect on job demands and psychological health in workers [36]. The results of a study conducted by Chen et al. showed that the safety climate can affect psychological stress and safety performance in workers [37]. The results of these studies confirm the findings of the present study and these findings have importance, particularly in petrochemical companies. Petrochemical companies have a large number of workers and health, safety, and environment (HSE) management systems so identification of the paths affecting the accident can be very helpful. The severity of accidents occurring in these companies is relatively high, which is associated with serious consequences. Also, most of the processes in these companies are controlled by humans, and the psychological conditions and perceptions of these people can be effective in preventing accidents [38]. Therefore, the results of the present study can be useful for these industries.

Of the safety climate dimensions, the variables of management’s safety priority, commitment, and competence and workers’ safety commitment possessed the highest direct and indirect effect coefficients on accident risk, respectively. Management’s safety priority represents the worker’s perception of management behavior on actions related to safety prioritization, even when production pressure is high. If managers are committed to performing the principles of safety with higher priority compared to other goals of the organization, the safe behaviors of employees can be improved. In a poor safety climate, managers and workers consider that safety responsibility is on each other rather than on themselves. However, a strong safety climate tends to be in an environment where managers and workers share the responsibility of safety with a converging perception of safety [21, 39]. Our findings suggest that high management’s safety priority, commitment, and competence are critical in creating a strong safety climate. Workers’ safety commitment is one of the other most substantial factors in improving the safety conditions in the workplace. If the worker is not committed to safety, unsafe behaviors, and human errors increase. So that the management cannot actualize the obligations [40, 41].

Among the job stress dimensions, the highest indirect effects were related to the variables of conflict at work, physical environment, workload and responsibility, and job control, respectively. The results of other studies also indicate the importance of conflict at work. The results of a study in Italy on female workers revealed that there is a relationship between conflict at work and occupational accidents, which is consistent with the results of this study [42]. The results of another study also showed that conflict at work compared to other organizational parameters had a stronger relationship to accidents and persons with more conflict experienced the accident with higher severity [43]. Based on the results of other studies, this factor has an important effect on people’s health in addition to job stress [44, 45]. The results of a study demonstrated that conflict at work can affect workers’ behavior and lead to accident occurrence [46]. In addition to this factor, there is an interaction between the employees and their surroundings workplaces. Therefore, the physical environment plays an important role in occupations because this factor influences several other factors of job stress. Appropriate design of the work environment can create feelings of comfort, safety, motivation, job satisfaction, and productivity in the workers. A job with a weak design for the physical environment is associated with increasing dissatisfaction, fatigue, and accidents [47, 48]. Moreover, the parameter of responsibility and workload is another important factor. If there is no balance between the tasks and employees’ abilities, people commit errors and accidents. The results of other studies confirm these findings [49, 50].

The limitations of this study include the lack of investigation of female workers and the lack of data analysis in different industries and job positions. Moreover, safety climate is a collective perception that should be measured at the group level [51] while the present study uses individual-level data. Another limitation of this study was the cross-sectional design for examining the causal relationships [52] although this study was performed based on past accident records. Therefore, it proposes that these limitations be considered in future studies. The effect of non-occupational stress due to family and community environments was not investigated, and the effect of demographic variables on the presented model was not studied. Therefore, it is recommended that these limitations be resolved in future studies. Finally, the effect of the proposed model on the number and severity of unsafe behaviors and the number and severity of work accidents were not considered, which authors will investigate in subsequent studies. Lastly, this study applies the self-report instruments as the data source, which may cause some bias in the results.

**Conclusion**The study found significant relationships between safety climate, job stress, and accident risk. Our results conclude that safety climate does not directly impact accident risk, but indirectly does so through job stress as a mediator. Job stress management/control offers the potential to mediate the relationships between safety climate and accident risk. In addition, other findings of this study could help industries, especially with safety challenges, exploit various tactics based on the identified/confirmed relationships among the various dimensions of safety climate, job stress, and accident risk.

## Data Availability

The datasets used and/or analyzed during the current study are available from the corresponding author on reasonable request.

## Abbreviations

SEM: Structural Equation Modeling
NOSACQ-50: Nordic safety climate questionnaire
GJSQ: generic job stress questionnaire.
